# First Genetic Detection and Characterization of Canine Parvovirus 2 in Botswana

**DOI:** 10.3390/v18070772

**Published:** 2026-07-14

**Authors:** Kebadire Tlotleng, Abdelkareem Abdallah, Solomon Stephen Ramabu, Davies Mubika Pfukenyi, Dhani Prakoso, Eman Ahmed Mohamed Anis

**Affiliations:** 1 Department of Veterinary Sciences, Faculty of Animal and Veterinary Sciences, Botswana University of Agriculture and Natural Resources, Private Bag 0027, Gaborone 999106, Botswana; aabdallah@buan.ac.bw (A.A.); ssramabu@buan.ac.bw (S.S.R.); dpfukenyi@buan.ac.bw (D.M.P.); 2 Pennsylvania Animal Diagnostic Laboratory System-New Bolton Center, Department of Pathobiology, University of Pennsylvania School of Veterinary Medicine, Kennett Square, PA 19348, USA; dprakoso@vet.upenn.edu

**Keywords:** Canine parvovirus, phylogenetic analysis, canine parvovirus variants

## Abstract

*Canine parvovirus 2 (CPV 2)* is a highly contagious viral pathogen responsible for severe gastroenteritis in canine species worldwide. This study provides the first comprehensive molecular characterization of CPV 2 variants circulating in domestic dogs in Botswana. A total of 187 fecal samples from diarrheic dogs presented at veterinary clinics were tested for CPV 2 using an ELISA point-of-care test and molecular assays for confirmation. A CPV 2 prevalence of 62.0% was detected via ELISA, compared to 100% via qPCR. Genetic characterization using minor groove binder probe-based qPCR assays and sequencing of the *VP2 gene* revealed the predominance of the CPV 2c variant (99.1%), with the CPV 2a variant detected in one sample from Maun village. Amino acid sequence analysis of the *VP2 gene* identified a key mutation at position 426 (glutamic acid) characteristic of the CPV2c variants. Phylogenetic analysis showed clustering of the Botswana CPV 2c isolates with Nigerian, Ethiopian, Gabon, Egyptian, and Asian CPV2c variants, while the CPV 2a isolate formed a cluster with CPV 2a variants from Nigeria. These findings confirm the circulation of CPV2a and CPV2c variants in Botswana, with CPV2c as the predominant circulating variant. Continuous monitoring of emerging CPV2 variants is essential for effective disease control in Botswana.

## 1. Introduction

*Canine parvovirus 2* emerged in the late 1970s as a highly contagious pathogen causing severe gastroenteritis in domestic dogs and wild Canidae populations worldwide [1]. The virus is classified as canine parvovirus 2 (CPV 2) to differentiate it from the less virulent *canine parvovirus 1 (CPV1)* [2]. Clinical signs of CPV 2 infection include anorexia, vomiting, hemorrhagic diarrhea and dehydration, and in severe cases, hypovolemic shock and death, particularly in young animals [3,4,5].

CPV 2 belongs to the *Parvoviridae* family and is 98% genomically similar to the *Feline panleukopenia virus (FPLV)* [2,6,7]. The virus has an approximately 5.3kb single-stranded DNA genome with open reading frames encoding two non-structural proteins (NSP1 and NSP2) involved in replication and three capsid proteins (VP1, VP2, and VP3) [8,9]. VP2 is the major antigenic determinant, constituting 90% of the viral capsid [10].

CPV 2 undergoes rapid mutation, with substitution rates comparable to RNA viruses [11], and this phenomenon resulted in the emergence of three antigenic variants over the years: CPV 2a, which emerged around 1979 with five key mutations (M87L, I101T, A300G, D305Y, and V555I) in the VP2 protein from the original CPV 2 [10,12,13]; CPV 2b, a new variant that emerged in 1980, with two additional amino acid changes in the VP2 protein (N426D and I555V); and CPV 2c, a variant found in 2000 with a single amino acid change (D426E), which was first reported in Italy [11,13]. Today, CPV 2a, 2b, and 2c are globally distributed, with variable prevalence across regions [5,14,15,16]. In 1990, the Ser297Ala mutation appeared in VP2 of CPV 2a and CPV 2b, and these were designated as new CPV 2a and new CPV 2b, respectively [17,18].

Vaccination remains the most effective strategy for CPV 2 control [19,20], with attenuated vaccines providing strong and long-lasting immunity. Despite widespread availability and use of these vaccines for CPV 2 prevention, CPV 2 cases in vaccinated dogs continue to be recorded globally, raising concerns about vaccine efficacy against emerging variants [21,22,23,24,25].

In Africa, a few studies have described the circulation of CPV 2 variants in countries such as Nigeria, South Africa, Namibia, Ethiopia, Zambia, Mozambique, Morocco, Tunisia, Egypt, and Gambia [8,26,27,28,29]. While serological evidence of CPV 2 has been documented in Ghana [30], Zimbabwe [31], Cape Verde [32] and Botswana [33], few studies have molecularly characterized the virus in these regions.

To date, limited information is available on the prevalence, distribution, and genetic characterization of CPV 2 variants circulating in Botswana. The current study presents the first comprehensive molecular analysis of CPV 2 variants in domestic dogs in Botswana. These findings aim to inform surveillance efforts, guide vaccine policy, and support improved control strategies for CPV 2 in Botswana and the Southern African region.

## 2. Materials and Methods

### 2.1. Study Design and Sample Collection

**Figure 1:**
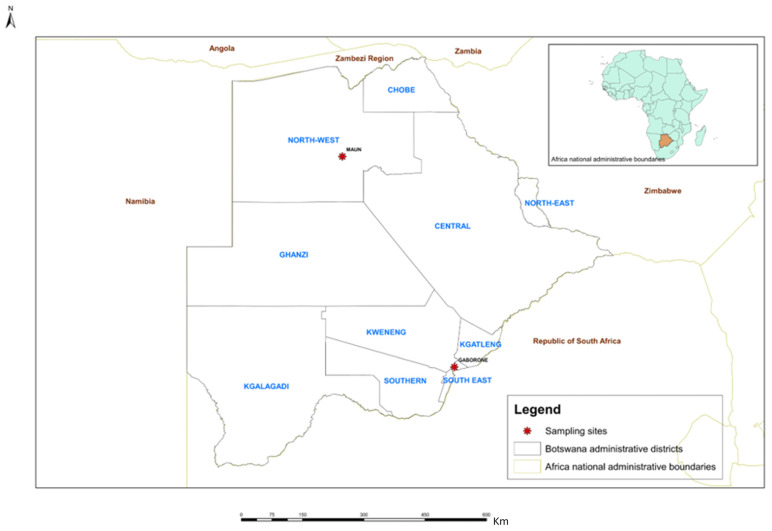
Map of Botswana indicating veterinary clinics and sampling sites.

The study was conducted in Botswana and approved by the Bioethics Committee at the Faculty of Animal and Veterinary Science, Botswana University of Agriculture and Natural Resources (BUAN-AEC-2022-07). A total of eight veterinary clinics across Botswana were selected to take part in this study: seven of these clinics were in and around the capital city, Gaborone, situated in the southeastern part of the country, with a population of 250,000 inhabitants [34], while one veterinary clinic was located in Maun village in the northwestern part of the country, with a population of around 80,000 inhabitants [34], as indicated in Figure 1.

Dogs presenting with gastrointestinal disorders from participating veterinary clinics were enrolled in the study with consent from their owners. An initial clinical examination of individual cases was conducted at the respective study sites. Demographic dog data, including sex, age, breed, origin, vaccination history, and number of dogs per household, were obtained using a pre-designed form. Dogs presenting clinical symptoms of vomiting, watery or hemorrhagic diarrhea, dehydration, anorexia, and foul smell were exclusively selected by the admitting veterinary clinicians. These dogs (*n* = 187) were subjected to an initial screening test against CPV 2 using the antigen capture ELISA test (SNAP Parvo, IDEXX Laboratories, Westbrook, ME, USA) following the manufacturer’s instructions, and results were recorded using forms.

Fecal samples were collected from the rectum of each suspected case using sterile swabs and stored at −80 °C until shipped on dry ice to the Pennsylvania Animal Diagnostic Laboratory System, New Bolton Center, School of Veterinary Medicine, University of Pennsylvania, for further testing. Dogs sampled were classified as vaccinated (*n* = 52), partially vaccinated (*n* = 34), not vaccinated (*n* = 98), or of unknown vaccination status (*n* = 3). The partially vaccinated dogs were those that received a single dose and did not get subsequent booster shots. CPV 2 vaccines commonly used in Botswana are imported from South Africa, including Vanguard Plus 5 Zoetis, Santon, 2196, South Africa, Nobivac^®^ DHPPi Intervet Pty LTD, Spartan, 1619, South Africa and Canigen DHPPi Virbac, SA Reg Number G2839, Santon, South Africa. Nobivac^®^ Puppy DP PLUS, manufactured by Intervet Pty LTD, Spartan, 1619, South Africa, is also routinely used as primary vaccination shots for puppies against parvovirus.

### 2.2. DNA Extraction and Real-Time PCR

Fecal swabs were suspended in 1 mL of phosphate-buffered saline (PBS, pH 7.4) in 1.5 mL tubes and clarified by centrifugation at 4000× *g* for 5 min to collect the supernatant. DNA from the supernatant was extracted using QIAamp Fast DNA Stool Mini Kit (Qiagen, Germantown, TN, USA) following the manufacturer’s instructions. For quality control, VetMAX™ Xeno™ Internal Positive Control (IPC) DNA (Thermo Fisher, Waltham, MA, USA) was added to evaluate the extraction process and monitor the presence of any inhibitors. The extracted DNA was stored at −80 °C for further analysis.

All samples were subjected to real-time PCR (qPCR) using previously published CPV 2 primers and a probe set [35]. Real-time PCR was carried out in a 25 µL reaction volume consisting of 12 µL of the VetMAX™ qPCR master mix (Thermo Fisher, Waltham, MA, USA), 1.25 µL of each primer (10 µM), 0.6 µL of the probe (10 µM), 1 µL of VetMAX™ Xeno™ Internal Positive Control DNA (Thermo Fisher, Waltham, MA, USA), 1.4 µL of nuclease-free water, and 7 µL of the DNA template. Amplification was performed on an Applied Biosystems 7500 real-time PCR instrument (Applied Biosystems, Waltham, MA, USA). The cycling conditions were as follows: 95 °C for 10 min as an initial denaturation step, followed by 40 cycles of 95 °C for 15 s and 60 °C for 1 min.

To determine the CPV 2 variants circulating in Botswana, all samples were further characterized using two previously published MGB probe-based multiplex qPCR assays specifically designed for the detection of CPV 2a /2b and CPV 2b /CPV 2c [36] as shown in Table 1.

The total reaction volume for the CPV2a/2b multiplex qPCR was 25 µL, consisting of 12.5 µL of Vetmax qPCR buffer, 1.25 µL each of forward and reverse primers for CPV 2 a/b (10 µM), 0.6 µL each of the CPV 2a and CPV 2b1 probes (10 µM), 1.8 µL of nuclease-free water, and 7 µL of the DNA template. A similar reaction mixture was used for the CPV 2b/c multiplex qPCR, with 1.25 µL each of forward and reverse primers for CPV 2b/c, and 0.6 µL each of CPV 2b2 and CPV 2c probes (10 µM). The reactions were conducted on an Applied Biosystems 7500 real-time PCR machine under the following conditions: 95 °C for 10 min followed by 40 cycles of 95 °C for 15 s and 60 °C for 1 min.

### 2.3. VP2 Gene Sequencing

A subset of samples (*n* = 35) was selected for full-length *VP2 gene* sequencing to enable genetic comparisons and further phylogenetic analyses. The selected samples originated from various veterinary clinics across different regions, including cohorts with different vaccination statuses (vaccinated, partially vaccinated, and non-vaccinated animals), CPV 2 variants, and a diverse range of Ct values. The full-length *VP2 gene* was amplified by three independent overlapping PCR assays using previously published primers, as shown in Table 2 [37]. Each PCR reaction was conducted in a final volume of 25 µL, consisting of 12.5 µL of Takara Ex-Taq DNA polymerase enzyme master mix (Takara Bio, San Jose, CA, USA), 0.4 µM of each primer, 8 µL of DNase RNase-free water, and 2.5 µL of the DNA template. Amplifications were carried out on SimpliAmp PCR thermocycler instrument (Applied Biosystems, Waltham, MA, USA) using the following cycling conditions: 94 °C for 10 min followed by 40 cycles of 95 °C for 30 s, 50 °C for 1 min, and 72 °C for 1 min with a final extension at 72 °C for 7 min. The PCR products were analyzed using electrophoresis in a 1.4% agarose gel containing ethidium bromide, and their clean-up was performed using ExoSAP-IT (Fisher Scientific, Waltham, MA, USA) according to the manufacturer’s protocol prior to submission for sequencing. The purified products were sent to a commercial company (Eurofins Scientific, Lancaster, PA, USA) for Sanger sequencing.

VP 2 sequence assembly and editing were performed using Geneious version 2023.1 software (http://www.geneious.com, accessed on 17 April 2024), with which the contigs were aligned using the MAFFT algorithm. Low-quality read ends were trimmed, and forward and reverse reads were aligned to generate a single contig, with complete identity required across the overlapping region. The resulting 1755 bp VP2 sequence was manually inspected for ambiguous bases and internal stop codons upon translation.

A total of 21 nucleotide sequences obtained from the study were submitted to GenBank for allocation of accession numbers for future reference. VP2 amino acid predictions were aligned and analyzed in MEGA 11 [38] for CPV 2 variant typing. The obtained sequences were translated into proteins, and comparisons were made with known reference isolates and common commercial CPV 2 vaccine variants available in GenBank; these results were summarized in Tables 5 and 6.

Phylogenetic trees were constructed using Bayesian Markov Chain Monte Carlo (MCMC) in Bayesian Evolutionary Analysis by Sampling Trees (BEAST X v10.5.0) [39] with a chain length of 50,000,000 and sampling at every 5,000,000 generations. The analysis utilized a fixed molecular clock, the Hasegawa, Kishino, and Yano (HKY) model, with gamma-distributed rates of variation among sites and a proportion of invariable sites (G+I). Tracer v1.7.2 [39] was used to visualize results and confirm chain convergence. Every parameter had an effective sample size (ESS) > 500, implying adequate sampling. Tree Annotator v10.5.0 [39] was utilized to choose the maximum clade credibility tree after a 10% burn-in. Posterior probabilities >90% were deemed statistically significant according to BEAST X v10.5.0. [39], and the trees were visualized using Fig Tree version 1.4.4 software (http://tree.bio.ed.ac.uk/software/figtree, accessed on 14 February 2026).

Two phylogenetic trees were constructed. The first was based on partial VP2 sequences (631basepairs) from this study and reference sequences obtained from GenBank (NCBI: http://www.ncbi.nlm.nih.gov, accessed on 14 February 2026), representing CPV 2 variants from Africa and the vaccine strains. The second tree was based on full-length VP2 sequences from this study and reference sequences from Africa, other regions of the world, and vaccine variants obtained from GenBank. Sequences were labeled as follows: GenBank accession number, antigenic variant, country, and year of sample collection.

### 2.4. Statistical Analysis

The ELISA and qPCR test results were paired proportions/percentages and therefore compared using McNemar’s online test calculator (https://www.medcalc.org/calc/mcnemar.php, version 23.3.5; accessed on 13 August 2025). The Kappa statistic was used to quantify the degree of agreement between the two tests. For the ELISA test results, comparisons among the different categories were performed using the statistical package EpiCal 2000 (available online at https://epicalc-2000.software.informer.com/, version 2; accessed on 13 August 2025). The percentage difference between the categories was assessed (analyzed by the chi-squared test for proportions), and *p*-values less than 0.05 were considered statistically significant. Since all samples were positive according to the qPCR, no comparisons were made between the different categories for the qPCR test results.

## 3. Results

The results from the questionnaire, point-of-care (ELISA), and real-time PCR tests are summarized in Table 3.

A total of 187 clinical cases were examined in the study, with most cases observed in young dogs aged 0-3 months (61%) and in male dogs (51.9%). Unvaccinated dogs comprised the majority at 52.4%, 68.4% of which were exotic breeds. Notably, 78.6% of cases from multi-dog dwellings accounted for over 70% of the total cases.

Overall, the positivity rate for the qPCR (100%) was significantly (X^2^ = 69.01, *p* < 0.0001) higher than that of the ELISA test (62%), and the results were similar for all dog categories. While qPCR showed consistently higher sensitivity across all tested groups, ELISA detected significantly more positives among dogs from multi-dog dwellings (66%) compared to single-dog dwellings (7.5%, X^2^ + 4.56, *p* < 0.033), with no significant (X^2^ < 3.1, *p* > 0.05) variation in the positivity rates of the ELISA results for the other categories.

### 3.1. Real-Time PCR

All samples tested were confirmed positive for CPV 2 by qPCR. A wide range of Ct values were observed (Table 4), with a greater number of unvaccinated and partially vaccinated dogs exhibiting low Ct values (<20), indicating high viral loads. Elevated viral loads are often associated with increased disease severity and progression.

### 3.2. Genotyping Using MGB Probe-Based Multiplex qPCR Assays

MGB probe-based multiplex qPCR assays were carried out to determine the CPV 2 genotypes present in the samples. The CPV 2c strain was detected in all but one sample, which was identified as CPV 2a and originated from Maun village in the northern part of the country (Supplementary Table S1).

### 3.3. VP2 Gene Sequence Analysis

A total of 35 samples were subjected to Sanger sequencing of the full-length *VP2 gene*, of which 20 yielded full-length VP2 sequences, and others yielded partial sequences of the gene. A total of 21 sequences were submitted to GenBank and were allocated accession numbers PX920305 to PX920325 (Supplementary Table S2). All sequences were characterized as CPV 2c displaying a glutamic acid (E) residue mutation at position 426 of the *VP2 gene* amino acid sequence, except one sample, J1 strain accession number PX920325, which was characterized as CPV 2a, displaying asparagine (N) at position 426. A unique mutation among Botswana samples was recorded in the Botswana BC1 strain (accession number PX920306) at amino acid position 218, with leucine substituted for isoleucine. A total of eleven non-synonymous mutations were recorded in the *VP2 gene* across CPV 2c variants from Botswana samples compared to the reference strain (accession number M38245) (Table 5). The mutations include A5G, I101T, L218I, F267Y, S297A, A300G, D305Y, Y324I, Q370R, N375D and N426E. Mutations at positions 101, 300, 305, and 375 recorded in Botswana samples were also common across selected African reference variants (Table 5). Substitution F267Y was evident in all variants except South African (HQ602977 and HQ602985) and Ethiopian (OM937844) strains, and substitution Y324I was also evident in all variants apart from the South African (HQ602977 and HQ602985) variants. Similarly to Botswana strains, some African variants displayed substitution A5G and Q370R (Ethiopian OM937842; Nigeria MT840294; Gabon OP611195 and OP611196; Egypt OM100698 and OM100699), and CPV 2c (MT840291) from Nigeria also displayed substitution Q370R.

Comparisons of the deduced amino acid sequences for the *VP2 gene* from the Botswana samples, commercial vaccine strains, and selected reference strain M38245 from GenBank revealed unique variations in mutation sites and patterns as outlined in Table 6.

The vaccine strains showed variations in both the number and the pattern of mutations compared to Botswana variants and the reference strain from the USA (accession number M38245). Vaccine strains with unique mutation sites not shared with Botswana strains include Vanguard Pfizer (accession number EU914139) with six mutations (positions T44A, I219K, C270V, N375E, Q386R, and Y573F) and only one similarity to Botswana strains at residue N375D; Merial BI/Parvodog (accession number FJ011097) with four unique mutations (T44A, K271R, V316I and K570E); Nobivac Intervet (accession number FJ01011098) with three mutations (K271R, V316I and K570T); and MSD-AH/Nobivac (accession number MJ197846) with one mutation (I219V). These last three vaccines did not share any mutation similarity with the Botswana variants. The Duramen Zoetis (accession number FJ222822) vaccine recorded the highest number of mutation sites at nine (M87L, I101T, S297A, A300G, D305Y, N321K, N375D, N426D, and K570E), seven of which (M87L, I101T, S297A, A300G, D305Y, N375D, and N426D/E) were similar to Botswana strains. Quantum Sharing (accession number GU212739) had the second highest number of mutations at eight (S35F, V38G, T44A, P207S, N375E, N426D, and Y573F), with only two mutations (N375E/D and N426D/E) being similar to the Botswana variant mutant sites.

### 3.4. Phylogenetic Analysis

**Figure 2:**
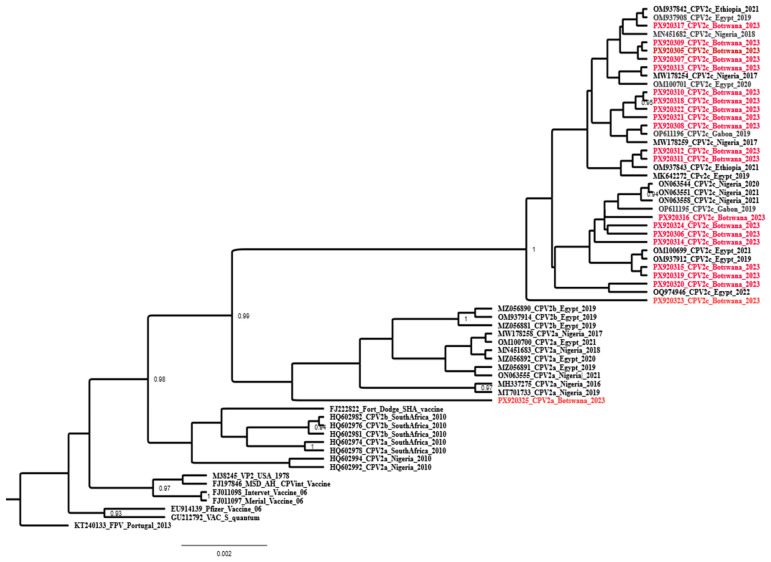
A phylogenetic tree based on partial VP2 gene sequences of CPV 2 generated using BEAST. The 21 sequences in red indicate variants circulating in Botswana. African and vaccine variant reference sequences were obtained from GenBank (http://www.ncbi.nlm.nih.gov, accessed on 14 February 2026) and contained information such as accession number, variant type, country of origin, and date of sampling.

A phylogenetic tree was generated using Bayesian Evolutionary Analysis by Sampling Trees (BEAST), and a comparison was performed between Botswana CPV 2 sequences (21 sequences) and reference sequences obtained from GenBank, including representative sequences from African and vaccine strains for CPV 2 (Figure 2). Almost all Botswana CPV 2c samples (20 sequences) clustered together with other African CPV 2c variants, mainly Nigeria, Egypt, Gabon, and Ethiopia, but only one sample accession number, PX920325 (J1 strain), clustered with CPV 2a from Nigeria. The vaccine variants clustered among themselves, except for the Fort Dodge SHA vaccine, formulated based on the CPV 2b variant, which clustered with South African CPV 2b sequences.

**Figure 3:**
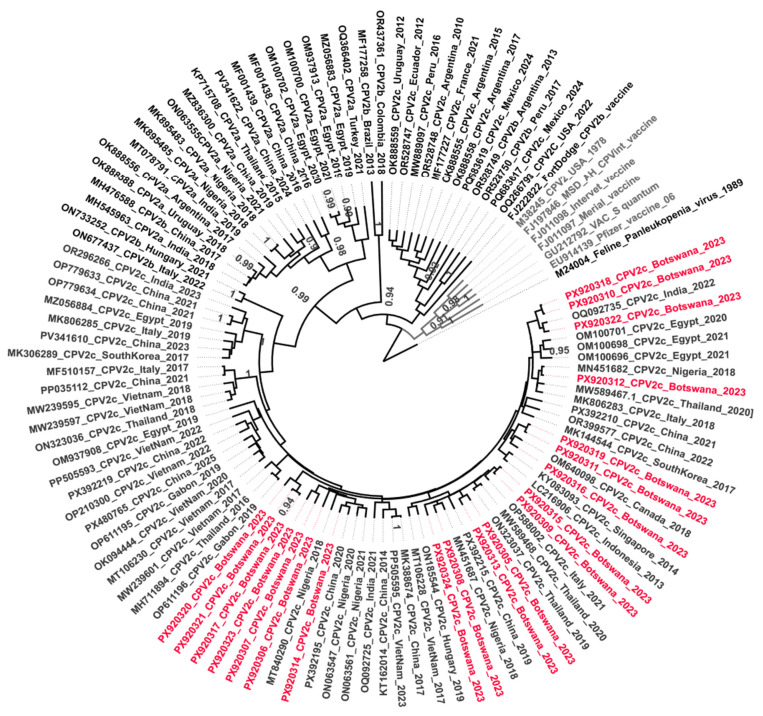
A phylogenetic tree based on the full-length VP2 sequence generated using BEAST X v10.5.0. Botswana CPV 2c sequences are marked red. Reference sequences from other global regions and vaccine variants retrieved from GenBank (http://www.ncbi.nlm.nih.gov, accessed on 14 February 2026). Feline panleukopenia (M24004) is an outgroup.

A total of 93 full-length CPV 2 *VP2 gene* reference sequences obtained from GenBank, representing global isolates, were compared with CPV 2c VP2 sequences from Botswana that were generated in this study (Figure 3). The phylogenetic tree showed tight clustering of Botswana CPV 2c variants with sequences from African CPV 2c and Asian CPV 2c variants.

## 4. Discussion

The present study describes the first detection and genetic characterization of CPV 2 circulating in domestic dog populations in Botswana. In this study, suspected CPV 2 cases from eight veterinary clinics across Botswana were initially screened by ELISA and confirmed by qPCR. The majority of cases (93.9%) occurred in dogs aged 0 to 6 months, consistent with findings from Egypt [41], Morocco [26] and Nigeria [43], where CPV 2 prevalence rates of 84.6%, 85.5%, and 93.3%, respectively, were reported in dogs of similar ages. This observation supports the notion that CPV 2 is predominantly a disease of younger animals [44] likely attributed to waning maternal-derived antibodies between 4 and 16 weeks of age, rendering puppies vulnerable to infection [45].

Multiple-dog households comprised the majority of CPV 2 cases, constituting 78.6% of cases detected in this study, and were identified as a risk factor facilitating the spread of the disease. Overcrowding has been documented as a potential risk factor for CPV 2 infection, especially in boarding kennels [42,46]. Exotic breeds were overrepresented with 68.4% cases, perhaps owing to sentimental care provided by their owners and being most likely to be sent to animal health care compared to local breeds. Overall, age, household density, and breed were the main factors significantly associated with CPV 2 infection, consistent with previous reports [42,47].

A high positivity rate was observed with qPCR (100%), compared to 62% positivity for ELISA, demonstrating the superior sensitivity of qPCR and its ability to detect low viral loads, even in early infection [48,49,50]. In contrast, ELISA is more prone to false-negative results [5,51], partly due to antigen sequestration forming immune complexes and its relatively high detection threshold requirement of approximately 10^6^ viral copies per milligram of feces [50,52]. The high PCR positivity among tested diarrheic dogs is consistent with reports from other regions, including Morocco [26] and Nigeria [43]. Locally, high seroprevalence has also been documented by Thompson et al. [33] in dogs in Maun, suggesting that the virus is endemic in Botswana. However, the high positivity is likely attributed to the cross-sectional design of the study, which focused exclusively on symptomatic dogs. Additionally, socioeconomic factors may delay owners from seeking veterinary care on time, leading to an overrepresentation of severely affected cases presented at veterinary clinics.

In this study, 47.3% of CPV 2 positive cases were from vaccinated or partially vaccinated dogs, while 52.3% were from unvaccinated animals. Similar trends have been reported in China, Morocco, Italy, and Australia, where 48.42%, 40.6%, 32.5% and 3.3% CPV 2-positive cases, respectively, were reported in vaccinated dogs [26,53,54,55]. These findings highlight the widespread occurrence of vaccine failure among vaccinated animals globally. Improper vaccination protocols, poor handling or maintenance of the vaccine cold chain, and use of vaccines with suboptimal cross-protection against circulating variants cannot be ruled out as potential causes of vaccine failure observed in these studies. The low qPCR Ct values (<20) observed in unvaccinated animals indicate higher viral loads, consistent with previous studies showing that unvaccinated or partially immunized dogs tend to exhibit more severe clinical signs and higher mortality rates compared to fully vaccinated animals [20,56].

Molecular genotyping utilizing MGB probe-based multiplex qPCR assays has identified CPV 2c as the predominant variant circulating in Botswana, with only a single CPV 2a variant detected in Maun village. The robustness of probe-based qPCR for CPV 2 differentiation is well established [26,36]. Sequencing of the *VP 2 gene* corroborated the qPCR findings and confirmed mutations at amino acid position 426, where asparagine was substituted for glutamic acid, thereby classifying the Botswana variants as CPV 2c. This N426E mutation occurs within the VP2 epitope A region comprising amino acid residues 93, 222, 224, and 426 [57,58], which is responsible for the emergence of antigenic variants of CPV-2a, CPV 2b, and CPV2c. Furthermore, mutations at amino acid positions 299 and 300–305, located within epitope B on the threefold axis of the CPV2 capsid structure [58,59], are known to be responsible for virus host range and antigenicity, as these residues are exposed on the viral surface. The Botswana CPV 2c variants exhibit a mutation at position 300 (A300G), which is commonly reported among CPV 2c variants [60]. Additionally, the substitution of tyrosine (Y) with aspartate (D) at position 305 was observed in the Botswana CPV 2c variants, which may contribute to antigenic differences, although its impact is likely modulated by interactions with other residues within the capsid.

Additionally, non-synonymous substitutions were observed in Botswana variants when compared with reference vaccine strains from GenBank (Table 6). This is expected, as currently available vaccines are derived from the earlier CPV2 or CPV2b variants and therefore show genetic divergence from the more recently emerged CPV 2c variants, driven by the high evolutionary rate of CPV 2 [61,62]. Through antigenic drift, three newly acquired mutations in the *VP2 gene*, F267Y, Y324I, and T440A, have been implicated in vaccine failure [40,63]. The Botswana variants exhibit the F267Y and Y324I mutants, associated with host adaptation and altered host range, respectively [40], but they lack the T440A mutation, which has been more strongly linked to changes in antigenicity [55,64].

Although previous studies have demonstrated cross-protection of CPV 2 and CPV 2b-based vaccines against heterologous variants, including CPV2c [20,62,65,66,67], incorporation of homologous CPV 2c variants into new future vaccine formulations has been proposed to enhance vaccine efficacy. Newer vaccines incorporating emerging genetic variants such as CPV 2c are currently under development [68], and they provide improved vaccine efficacy and overcome the interference of maternal antibodies following primary vaccination.

Amino acid substitutions previously reported in Asian CPV 2c variants, including A5G, F267Y, Y324I, and Q370R, were also detected in the Botswana variants [69,70]. Importantly, while F267Y and Y324I were discussed above in the context of their impact on vaccine failure, their presence in this dataset also supports the broader molecular similarity of Botswana variants to the globally circulating CPV 2c lineage. The A5G mutation is located at the N-terminus of VP2, while the Q370R is located around the protein-binding pocket, and both are believed to be associated with changes in host range and the protein-binding capacity of the virus [71]. The presence of these mutations in Botswana CPV 2c variants suggests that CPV 2c circulating in Southern Africa shares molecular features with globally emerging variants, potentially reflecting viral adaptation. A unique mutation at amino acid position 218 (L218I) was identified in the Botswana BC1 strain (accession number PX920306). Overall, mutations observed in Botswana CPV 2c variants are predominantly located outside the traditionally mapped epitope regions of the CPV2 capsid, which are primary targets for neutralizing antibodies against the virus [72]. Therefore, these substitutions are unlikely to affect the cross-protective efficacy of existing vaccines against the currently circulating CPV2c variant. Some studies have previously demonstrated that CPV 2b and CPV 2c variants are mutually cross-neutralized by antisera raised against either variant, indicating that the 426E substitution does not confer immune escape properties [67,73]. However, given the limited data on in vitro cross-neutralization using Botswana isolates, continued molecular surveillance of circulating CPV 2 variants is recommended. Future studies employing virus neutralization assays to directly assess vaccine-induced antibody cross-reactivity against Botswana CPV 2c field strains are warranted.

A phylogenetic analysis revealed that the Botswana CPV 2c variants formed a monophyletic clade with CPV 2c reference strains from other African countries, including Gabon, Nigeria, Egypt, and Ethiopia (Figure 2), as well as from Asian countries like India, Vietnam, China, Indonesia, and Thailand (Figure 3). This supports the hypothesis regarding the regional and transcontinental spread of this Asian CPV 2c lineage into Africa [8,28,74] likely facilitated by increased trade and pet movement between the two continents.

The single CPV 2a variant from Botswana clustered with CPV 2a variants from Nigeria, which may be attributed to trade or movement of goods between the two countries.

The study had some limitations as CPV 2 cases were not sampled from all districts of the country, which would have provided a more comprehensive epidemiological map of the disease. This was constrained by the limited resources available to undertake such an expanded study. Additionally, the research mainly focused on sick dogs presented to veterinary clinics. This limitation affected the statistical analysis, as all samples tested positive when using qPCR. The absence of negative qPCR results made analytical risk factors difficult to compute and necessitated the use of ELISA results instead. It is therefore advised that the results of the risk factors be interpreted with caution and treated as preliminary findings from this study. Future epidemiological studies should include negative samples to enable more robust estimation of true prevalence and associated risk factors of the disease in the country.

For phylogenetic analysis, most African sequence variants had short partial VP2 sequences available in GenBank, which affected molecular phylogenetic tree reconstruction. A whole-genome sequence of the virus would provide deeper insight into viral evolution as well as phylogenetic and global phylogeographic relationships of the pathogen.

## 5. Conclusions

This study provides insights into the presence and genetic diversity of CPV 2 variants in Botswana. The findings confirm the active circulation of CPV 2a and CPV 2c variants within the country. Notably, CPV 2c has emerged as the predominant variant associated with most cases of CPV 2. These findings have significant implications for CPV 2 control in Botswana, underscoring the critical importance of routine molecular surveillance to monitor viral evolution and inform future diagnostic and vaccination strategies. This study also highlights the need to improve vaccination coverage, particularly in young dogs and multi-dog households, to mitigate viral spread and disease severity. However, risk analysis findings should be interpreted with caution and considered preliminary, as they were based on ELISA results following the absence of negative qPCR samples.

Future work may cover a comprehensive nationwide epidemiological investigation involving both domestic and wild carnivores, which is essential for fully comprehending the diversity and geospatial distribution of CPV 2 in Botswana. Also, future studies employing virus neutralization assays to directly assess vaccine-induced antibody cross-reactivity against Botswana CPV 2c field strains are warranted.

## Figures and Tables

**Table 1:** Real-Time PCR MGB probes and set of primers for characterization of CPV 2 variants, [36].

Primer/Probe	Sequences	Position	Amplicon Size (bp)	Reference
CPV 2a/b Forward	5′ AGGAAGATATCCAGAAGGAGATTGGA 3′	1719–1744	93	[36]
CPV 2a/b Reverse	5′ CCAATTGGATCTGTTGGTAGCAATACA 3′	1785–1811		
CPV 2a probe	VIC-CTTCCTGTAAATGA-MGB	1765–1783		
CPV 2b_1_	FAM-CTTCCTGTAACAGATGA-MGB	1765–1783		
CPV 2b/c Forward	5′ GAAGATATCCAGAAGGAGATTGGATTCA 3′	1721–1748	150	[36]
CPV 2b/c Reverse	5′ ATGCAGTTAAAGGACCATAAGTATTAAATATATTAGTATAGTTAATTC 3′	1823–1870		
CPV 2c probe	VIC-CCTGTAACAGAAGATAA-MGB	1202–1219		
CPV 2b -probe	FAM-CCTGTAACAGATGATAAT-MGB	1768–1785		

**Table 2:** Primers used for amplification of the full-length VP2 gene of CPV 2.

Primers Pairs	Sequence	Position	Amplicon Size (bp)	Reference
555 Forward	CAGGAAGATATCCAGAAGGA	4003–4022	583	[37]
555 Reverse	GGTGCTAGTTGATATGTAATAAACA	4561–4585		
3381 Forward	CCATGGAAACCAACCATACC	3381–3400	717	
4116 Reverse	AGTTAATTCCTGTTTTACCTCCAA	4093–4116		
2655 Forward	AAAAAGAGACAATCTTGCACCA	2655–2675	837	
3511 Reverse	TGAACATCATCTGGTCTACC	3489–3511		

**Table 3:** Summary of ELISA and qPCR results for the samples analyzed.

				Test			
		Clinical Cases		ELISA		qPCR	
Variable	Level	Total	% of Total	No. Positive	% Positive	No. Positive	% Positive
	All	187	-	116	62.0	187	100
Age	0–3 months	114	61.0	75	65.8 ^a^	114	100
	4–6 months	62	33.2	34	54.8 ^a^	34	100
	>6 months	11	5.9	7	63.6 ^a^	11	100
Sex	Male	97	51.9	64	66.0 ^a^	97	100
	Female	90	48.1	52	57.8 ^a^	90	100
Vaccination	Vaccinated	52	27.8	33	63.5 ^a^	52	100
	Unvaccinated	98	52.4	58	59.2 ^a^	98	100
	Partially vaccinated	34	18.2	24	70.6 ^a^	34	100
	Unknown	3	1.6	1	33.3 ^a^	3	100
Dwelling	Multi-dog	147	78.6	97	66.0 ^a^	147	100
	Single	40	21.4	19	47.5 ^b^	40	100
Breed	Mixed	48	25.7	31	64.6 ^a^	48	100
	Indigenous	11	5.9	8	72.7 ^a^	11	100
	Exotic	128	68.4	77	60.2 ^a^	128	100

Figures in a column with a different superscript for the same variable under the ELISA test are significantly different at *p* < 0.05.

**Table 4:** Range of Ct values obtained by qPCR.

Ct Values	Vaccinated	Partially Vaccinated	Not Vaccinated	Unknown	Total
<10	1	1	4	0	6
10–20	29	24	54	2	109
20–30	17	4	37	1	59
>30	5	3	5	0	13
	52	32	100	3	187

**Table 5:** VP2 amino acid substitutions in Botswana CPV 2c variants relative to reference strain M38245 and African reference strains from GenBank.

Accession Number	Strain	Variant	Origin	Year	5	38	101	218	267	297	300	305	324	370	375	392	426	440	447	References
M38245	CPV b	CPV2	USA	1978	A	V	I	L	F	S	A	D	Y	Q	N	G	N	T	I	[40]
HQ602977	101-10SA 2010	CPV-2a	South Africa	2010	-	-	T	-	-	-	G	Y	-	-	D	-	-	-	-	[27]
OM937844	18a	CPV-2a	Ethiopia	2021	-	-	T	-	-	A	G	Y	I	-	D	-	-	-	-	[8]
MK895483	IZSSI_PA1464/19_iduV1	CPV2a	Nigeria	2018	-	-	T	-	Y	A	G	Y	I	-	D	-	-	A	-	[8]
MZ056883	EGY-FVMVL-21/2019	CPV-2a	Egypt	2019	-	E	T	-	Y	A	G	Y	I	-	D	-	-	A	-	[41]
HQ602985	8-10SA 2010	CPV-2b	South Africa	2010	-	-	T	-	-	N	G	Y	-	-	D	-	D	-	-	[27]
OM937842	16d	CPV-2c	Ethiopia	2021	G	-	T	-	Y	A	G	Y	I	R	D	-	E	A	-	[8]
MT840291	IZSSI-PA1464/19-idEVS5-TR-4A72	CPV 2c	Nigeria	2018	-	-	T	-	Y	A	G	Y	I	R	D	-	E	-	-	[42]
MT840294	IZSSI-PA1464/19-idYV7-TR-4A72	CPV-2c	Nigeria	2018	G	-	T	-	Y	A	G	Y	I	R	D	-	E	-	-	[42]
OP611196	Gab-9	CPV-2c	Gabon	2019	G	-	T	-	Y	A	G	Y	I	R	D	-	E	-	-	[29]
OP611195	Gab-10	CPV-2c	Gabon	2019	G	-	T	-	Y	A	G	Y	I	R	D	-	E	-	M	[29]
OM100698	EGY-FVMVL-21/2019	CPV 2c	Egypt	2021	G	E	T	-	Y	A	G	Y	I	R	D	V	E	-	-	[41]
OM100699	EGY_FVMVL-26/2019	CPV-2c	Egypt	2021	G	-	T	-	Y	A	G	Y	I	R	D	-	E	-	-	[41]
PX920305	BG-12	CPV-2c	Botswana	2023	G	-	T	-	Y	A	G	Y	I	R	D	-	E	-	-	Study
PX920306	BC-1	CPV-2c	Botswana	2023	G	-	T	I	Y	A	G	Y	I	R	D	-	E	-	-	Study
PX920307–PX920324 ^1^	Other Botswana Strains	CPV-2c	Botswana	2023	G	-	T	-	Y	A	G	Y	I	R	D	-	E	-	-	Study

^1^ Botswana isolates PX92037 to PX920324 (includes isolates BC-2, G-3, G-32, G-37, G-41, G-50, H-10, S-16, S-21, S-30, S-53, S-6, S-11, M-27, G-35, H-11, H-12, and M-2) exhibit identical VP2 amino acid profiles relative to the reference strain M38245. A dash (-) indicates identity with the amino acid residues and the reference strain M38245.

**Table 6:** VP2 amino acid substitutions in Botswana CPV-2c isolates compared with selected reference and vaccine strains.

NCBI GenBank No.	Strain	CPV Variant	Year	Origin	VP2 Amino Acid Substitutions Relative to M38245 ^1^
Reference and Vaccine Strains					
M38245	CPV-b	CPV-2	1978	USA	Reference Strain
M24003	CPV-15	CPV-2a	1984	USA	87L, 101T, 300G, 305Y, 375D
M74849.1	CPV-39	CPV-2b	1984	USA	87L, 101T, 300G, 305Y, 375D, 426D
MF177239.1	288-01	CPV-2c	2001	Italy	87L, 101T, 297A, 300G, 305Y, 375D, 426E
FJ222822	Duramune (Zoetis)	CPV-2b	2009	Italy	87L, 101T, 297A, 300G, 305Y, 321K, 375D, 426D, 570E
GU212792	Quantum (Schering)	CPV-2b	2010	Thailand	35F, 38G, 44A, 207S, 375E, 379V, 426D, 573F
EU914139	Vanguard (Pfizer)	CPV-2	2006	USA	44A, 219K, 270V, 375D, 386R, 573F
FJ011097	Merial (Parvodog)	CPV-2	2006	France	44A, 271R, 316I, 570T
FJ011098	Nobivac (Intervet)	CPV-2	2006	USA	219V, 316I, 570T
FJ197846	Nobivac (MSD-AH)	CPV-2	2007	South Korea	219V
Botswana Isolates (This Study)					
PX920305	Botswana/G-12	CPV-2c	2023	Botswana	5G, 87L, 101T, 267Y, 297A, 300G, 305Y, 324I, 370R, 375D, 426E
PX920306	Botswana/BC-1	CPV-2c	2023	Botswana	5G, 87L,101T, 218I, 267Y, 297A, 300G, 305Y, 324I,370R, 375D, 426E
PX920307	Botswana/BC-2	CPV-2c	2023	Botswana	5G, 87L, 101T, 267Y, 297A, 300G, 305Y, 324I, 370R, 375D, 426E
PX920308–PX920324 ^2^	Other Botswana Sample Strains	CPV-2c	2023	Botswana	5G, 87L, 101T, 267Y, 297A, 300G, 305Y, 324I, 370R, 375D, 426E

^1^ Amino acid substitutions are shown relative to the CPV2 reference strain M38245. ^2^ Isolates PX920308–PX920324 exhibited identical VP2 amino acid substitution patterns and were therefore grouped.

## Data Availability

The original contributions presented in this study are included in the article/Supplementary Materials. Further inquiries can be directed to the corresponding authors.

## Supplementary Materials

The following supporting information can be downloaded at https://www.mdpi.com/article/10.3390/v18070772/s1, Supplementary Table S1: Botswana sample distribution showing ELISA, real-time PCR results, and characterization of CPV 2 variants. Supplementary Table S2: Botswana CPV2 samples and their assigned accession numbers from GenBank.
