# Supplementary material for: First Genetic Detection and Characterization of Canine Parvovirus 2 in Botswana
Source: Viruses. 2026 Jul 14;18(7):772. doi: 10.3390/v18070772 (PMC13431485; doi:10.3390/v18070772)
Supplement: Supplementary file 1 [file viruses-18-00772-s001.zip › viruses-4355446-supplementary.pdf]

First Genetic Detection and Characterization of Canine Parvovirus 2 in Botswana

Kebadire Tlotleng [1,2,*], Abdelkareem Abdallah [1], Solomon Stephen Ramabu [1], Davies Mubika Pfukenyi [1], Dhani Prakoso [2] and Eman Ahmed Mohamed Anis [2,*]

[1] Department of Veterinary Sciences, Faculty of Animal and Veterinary Sciences, Botswana University of Agriculture and Natural Resources, Private Bag 0027, Gaborone 999106, Botswana; aabdallah@buan.ac.bw (A.A.); ssramabu@buan.ac.bw (S.S.R.); dpfukenyi@buan.ac.bw (D.M.P.)

[2] Pennsylvania Animal Diagnostic Laboratory System-New Bolton Center, Department of Pathobiology, University of Pennsylvania School of Veterinary Medicine, Kennett Square, PA 19348, USA; dprakoso@vet.upenn.edu

* Correspondence: ktlotleng@buan.ac.bw (K.T.); eanis@vet.upenn.edu (E.A.M.A.); Tel.: +267-71659694 (K.T.)

Supplementary material

Supplementary Table S1: Botswana Sample distribution showing ELISA, Real Time PCR results and characterization of CPV 2 variants by MGB probes.

Sample ID	Age (months)	sex	Breed	Origin	Clinical sings	Vaccination status	ELISA	Real-time PCR	CPV variant
S1	2	f	cross breed	Gaborone	V,Di,De,A,F	Not vac	Pos	26.53	CPV 2c
S2	2	f	cross breed	Gaborone	V,Di,De,A,F	Not vac	Neg	23.85	CPV 2c
S3	3	f	Rottweiler	Gaborone	V,Di,F	Not vac	Pos	19.62	CPV 2c
S4	2	f	cross breed	Gaborone	V,Di,De,A,F	Vacc	Pos	18.41	CPV 2c
S5	2	m	cross breed	Gaborone	V,Di,De,A,F	Not vac	Neg	23.24	CPV 2c
S6	2	f	Jack Russel Terrier	Gaborone	V,Di,De,A	Not vac	Neg	19.82	CPV 2c
S7	3	m	cross breed	Gaborone	V,Di,De,A,F	Not vac	Neg	29.02	CPV 2c
S8	4	f	cross breed	Gaborone	V,Di,De,A,F	Not vac	Neg	26.94	CPV 2c
S9	3	m	Jack Russel Terrier	Gaborone	V,Di,De,A,F	Not vac	Pos	16.08	CPV 2c
S10	3	f	Maltese	Gaborone	V,Di,A,F	Vacc	Pos	15.72	CPV 2c
S11	3	f	Boerboel	Kanye	V,Di,De,A,F	Vacc	Neg	21.40	CPV 2c
S12	3	f	Jack Russel Terrier	Gaborone	V,Di,De,A	Not vac	Neg	18.64	CPV 2c
S13	4	m	German Sheperd	Gaborone	V,Di,De,A,F	Vacc	Pos	16.05	CPV 2c
S14	6	m	Maltese	Gaborone	V,Di,De,A	Vacc	Neg	30.31	CPV 2c
S15	3	m	Boerboel	Gaborone	V,Di,De,A	Par vacc	Neg	26.77	CPV 2c
S16	3	f	German Sheperd	Gaborone	V,Di,De,A,F	Not vac	Neg	20.08	CPV 2c
S17	2	m	Jack Russel Terrier	Gaborone	V,Di,De,A,F	Par vacc	Pos	15.88	CPV 2c
S18	3	m	Rhodesian ridgeback	Gaborone	V,Di,De,A	Not vac	Neg	23.56	CPV 2c
S19	5	m	cross breed	Gaborone	V,Di,De,A,F	Not vac	Neg	19.29	CPV 2c
S20	3	f	Boerboel	Gaborone	V,Di,De,A,F	Not vac	Neg	21.23	CPV 2c
S21	3	f	cross breed	Gaborone	V,Di,De,A,F	Not vac	Pos	23.09	CPV 2c
S22	2	f	Jack Russel Terrier cross	Gaborone	V,Di,De,A,F	Not vac	Pos	27.87	CPV 2c
S23	2	m	Jack Russel Terrier	Gaborone	V,Di,De,A,F	Not vac	Neg	18.23	CPV 2c
S24	3	m	cross breed	Gaborone	Di,De	Not vac	Pos	24.29	CPV 2c
S25	3	f	cross breed	Gaborone	A	Not vac	Neg	19.89	CPV 2c
S26-1	3	f	Boxer	Gaborone	V,Di,De,A	Not vac	Pos	16.05	CPV 2c
S26-2	3	m	German Sheperd	Gaborone	V,Di,De,A	Not vac	Pos	16.41	CPV 2c
S27	3	m	cross breed	Gaborone	V,Di,De,A	Not vac	Neg	29.92	CPV 2c
S28	4	f	Jack Russel Terrier	Gaborone	A	Not vac	Neg	11.70	CPV 2c
S29	6	m	Pitbull	Gaborone	V,Di,De,A	Vacc	Pos	29.81	CPV 2c

S30	2	f	German Sheperd	Gaborone	V,Di,De,A,F	Vacc	Neg	21.61	CPV 2c
S31	2	m	German Sheperd	Gaborone	V,Di,De,A,F	Not vac	Pos	13.78	CPV 2c
S52	3	f	Maltese	Gaborone	V,Di,De,A,F	Par vacc	Pos	16.07	CPV 2c
S53	12	f	Maltese	Gaborone	V,Di,A,F	Par vacc	Neg	21.57	CPV 2c
S54	3	m	German Sheperd	South Africa	V,Di,A,F	Vacc	Pos	13.69	CPV 2c
S55	3	f	German Sheperd	South Africa	V,Di,De	Vacc	Pos	12.19	CPV 2c
S56	4	f	Jack Russel Terrier	Gaborone	V,Di,De	Not vac	Neg	22.35	CPV 2c
S57	3	f	cross breed	Gaborone	A	Not vac	Neg	16.98	CPV 2c
S58	4	m	Jack Russel Terrier	Gaborone	V,Di,De,A,F	Not vac	Pos	23.67	CPV 2c
S59	4	f	Jack Russel Terrier	Gaborone	V,Di,De,A,F	Not vac	Pos	15.30	CPV 2c
S60	3	f	cross breed	Gaborone	De,A	Vacc	Neg	17.90	CPV 2c
S61	3	f	Jack Russel Terrier	Gaborone	V,Di,De,F	Not vac	Pos	13.80	CPV 2c
S62	5	f	cross breed	Gaborone	Di,De,A	Not vac	Pos	25.93	CPV 2c
S70	4	f	Boerboel	Gaborone	V,Di,De,A,F	Not vac	Pos	18.46	CPV 2c
S71	3	m	German Sheperd	Gaborone	V,Di,De,A,F	Not vac	Pos	26.27	CPV 2c
S72	3	f	Boerboel	Gaborone	V,Di,De,A,F	Not vac	Neg	20.63	CPV 2c
S73	2	f	cross breed	Gaborone	V,Di,De,A,F	Vacc	Pos	13.61	CPV 2c
S74	2	m	cross breed	Gaborone	Di,A,F	Vacc	Neg	24.20	CPV 2c
S76	3	f	Jack Russel Terrier	Gaborone	V,Di,De,A,F	Not vac	Neg	17.27	CPV 2c
S77	3	m	Jack Russel Terrier	Gaborone	V,Di,De,A	Vacc	Neg	29.90	CPV 2c
S78	2	m	Maltese	Gaborone	V,Di,De,A	Vacc	Pos	15.24	CPV 2c
S79	3	m	Boerboel	Gaborone	V,Di,De	Par vacc	Pos	18.40	CPV 2c
S80	3	f	Maltese	Gaborone	V,Di,De,A	Not vac	Neg	14.09	CPV 2c
S81	4	f	Jack Russel Terrier	Gaborone	V,Di,De,A,F	Par vacc	Pos	15.43	CPV 2c
S82	2	m	mixed	Gaborone	V,Di,De,A,F	Not vac	Pos	35.10	CPV 2c
S83	2	m	Jack Russel Terrier	Gaborone	V,Di,De,A,F	Not vac	Pos	12.72	CPV 2c
S84	6	m	mixed	Gaborone	V,Di,De,A,F	Not vac	Neg	35.23	CPV 2c
S85	2	f	Maltese	Gaborone	V,Di,A	Not vac	Pos	22.26	CPV 2c
S86	3	m	Boerboel	Gaborone	Di,A	Not vac	Pos	13.44	CPV 2c
G1	2	m	Tswana	Gaborone	V,Di,De,A,F	Not vac	Pos	18.32	CPV 2c
G2	2	m	Tswana	Gaborone	V,Di,De,A,F	Not vac	Pos	15.79	CPV 2c

G3	6	f	Jack Russel Terrier	Gaborone	V,Di,De,A,F	Not vac	Pos	8.41	CPV 2c
G4	3	m	Bull terrier	Gaborone	Di,De,F	Not vac	Pos	14.94	CPV 2c
G5	5	f	Jack Russel Terrier	Gaborone	V,Di,De,F	Not vac	Neg	21.74	CPV 2c
G6	5	m	Maltese cross	Gaborone	V,Di,F	Not vac	Neg	19.28	CPV 2c
G6"	6	m	Jack Russel Terrier	Gaborone	Di,,F	Not vac	Neg	23.17	CPV 2c
G7	2	f	Rhodesian Ridgeback	Otse	V,Di,A	Vacc	Pos	9.17	CPV 2c
G8	4	f	Maltese	Gabs	V,Di,A,F	Vacc	Neg	8.85	CPV 2c
G9	7	m	Mixed	Molepolole	V	Not vac	Neg	17.55	CPV 2c
G10	2.5	f	Tswana	Gaborone	V	Par vacc	Pos	8.92	CPV 2c
G11	2.5	m	Mixed	Ruretse	No data	Par vacc	Neg	18.43	CPV 2c
G12	3	m	Pitbull	Gaborone	V,Di,De,A,F	Vacc	Pos	9.35	CPV 2c
G13	5	m	Maltese cross	Gaborone	V,Di,De	Vacc	Pos	10.43	CPV-2c
G14	2	m	Maltese cross	Gaborone	V	Not vac	Pos	13.55	CPV 2c
G15	4	m	German Shepherd	Molepolole	V	Not vac	Neg	19.73	CPV 2c
G16	4	m	Bull Mastiff	Mmopane	No data	Not vac	Pos	19.05	CPV-2c
G17	3	m	Maltese	Mogoditshane	V,Di,De,A	Not vac	Neg	15.15	CPV 2c
G18	4	m	Maltese cross	Gaborone	Di,De,A,F	Vacc	Pos	20.57	CPV 2c
G19	5	m	Tswana	Gaborone	V,Di,De,A,F	Par vacc	Pos	15.88	CPV 2c
G20	4	m	Maltese cross	Gaborone	No data	Not vac	Pos	23.77	CPV 2c
G21	3	m	Rottweiler cross	Gaborone	V,Di,De,A,F	Vacc	Pos	21.35	CPV 2c
G22	3	f	Jack Russel Terrier	Gaborone	V	Not vac	Pos	33.08	CPV 2c
G23	4	f	German Sheperd	Gaborone	V,De,A	Vacc	Pos	17.80	CPV 2c
G23"	4	m	Greyhound	Gaborone	V,Di,De,A	Not vac	Neg	18.41	CPV 2c
G24	3	m	Rottweiler	Gaborone	V,Di,F	Vacc	Pos	14.65	CPV 2c
G25	7	f	Tswana	Gaborone	V,Di,De,A,F	Not vac	Neg	23.43	CPV 2c
G26	2	f	Maltese cross	Gaborone	V,Di	Vacc	Neg	33.66	CPV 2c
G27	5	f	Bull Mastiff	Mmopane	No data	Par vacc	Neg	33.86	CPV 2c
G28	5	f	Chow chow	Gaborone	V,Di,F	Par vacc	Pos	16.89	CPV 2c
G29	9	f	Swiss Sheperd	Gaborone	V	Par vacc	Pos	23.13	CPV 2c
G30	13	f	Maltese cross	Gaborone	V,Di	Par vacc	Pos	17.06	CPV 2c

G31	2	f	Tswana	Mogoditshane	Di,F	Not vac	Neg	30.39	CPV 2c
G32	1	f	Jack Russel Terrier	Gaborone	V,Di,F	Not vac	Pos	21.08	CPV 2c
G33	4	m	Jack Russel Terrier	Gaborone	V	Vacc	Pos	17.32	CPV 2c
G34	3	f	Chow cross	Gaborone	V,Di,A	Par vacc	Pos	14.36	CPV 2c
G35	2	m	Pitbull cross	Tlokweng	V,Di	Par vacc	Pos	16.24	CPV 2c
G36	4	f	Rhodesian Ridgeback	Gaborone	V,Di	Vacc	Pos	15.07	CPV 2c
G37	2	m	German Sheperd	Gaborone	Di	Not vac	Pos	17.17	CPV 2c
G38	2	f	Tswana	Metsimotlhabe	V,Di,De,A,F	Not vac	Neg	27.29	CPV 2c
G39	3	m	Jack Russel Terrier	Gaborone	V,Di,De,A,F	Not vac	Pos	12.58	CPV 2c
G40	2	m	Maltese	Gaborone	V,Di	Not vac	Pos	10.67	CPV 2c
G41	3	f	Boerboel	Gaborone	V,Di,De,F	Not vac	Neg	21.02	CPV 2c
G42	4	m	Jack Russel Terrier cross	Gaborone	V, D	Vacc	Pos	16.26	CPV 2c
G43	4	m	Jack Russel Terrier	Gaborone	V	Vacc	Pos	17.22	CPV 2c
G44	3	f	Pitbull	Gaborone	V,Di,De,F	Vacc	Pos	11.74	CPV 2c
G45	4	f	Labrador	Gaborone	Di	Vacc	Neg	29.88	CPV 2c
G46	3	f	Jack Russel Terrier	Gaborone	V,Di,F	Vacc	Neg	29.45	CPV 2c
G47	4	m	Jack Russel Terrier	Gaborone	V,Di,De	Par vacc	Pos	19.69	CPV 2c
G48	5	f	Boerboel	Gaborone	V,Di,De,A	Not vac	Neg	22.96	CPV 2c
G49	5	m	Jack Russel Terrier	Gaborone	No data	Not vac	Neg	23.93	CPV 2c
G50	3	F	Tswana	Gaborone	V,Di,De	Not vac	Pos	11.93	CPV 2c
O1	2	m	Great Dane	Gaborone	A, F	Not vac	Pos	19.10	CPV 2c
O2	2	f	Great Dane	Gaborone	A,F	Not vac	Pos	16.54	CPV 2c
O3	3	f	Husky	Gaborone	A	Vacc	Pos	19.94	CPV 2c
O4	2	f	Maltese	Gaborone	Di,A,F	Par vacc	Pos	14.62	CPV 2c
O5	2	m	German Sheperd	Gaborone	V,Di,A	Not vac	Neg	20.44	CPV 2c
O6	2	f	Maltese	Gaborone	No data	Vacc	Pos	14.06	CPV 2c
O7	3	f	Rottweiler	Gaborone	A, F	Not vac	Pos	13.92	CPV 2c
O8	2	f	Jack Russel Terrier x Maltese	Gaborone	F	Vacc	Pos	16.99	CPV 2c
O9	3	m	mixed	Gaborone	No data	Not vac	Pos	15.63	CPV 2c
M1	2	F	Rhodesian Ridgeback	Oodi	Di	Vacc	Pos	14.79	CPV 2c
M2	3	m	Maltese	Ledumadumane	V,Di,A,F	Not vac	Pos	11.88	CPV 2c
M3	3	m	Swiss Sheperd	Gabs	V,Di,A	Vacc	Pos	21.36	CPV 2c

M4	2	m	Tswana	Oodi	V	Par vacc	Pos	18.98	CPV 2c
M5	6	f	Maltese	Phakalane	V,Di,De	Par vacc	Pos	16.62	CPV 2c
M6	5	m	Rottweiler	Phakalane	V,Di,De,A	Par vacc	Neg	11.30	CPV 2c
M7	6	f	Maltese	Phakalane	Di,De	Par vacc	Pos	17.10	CPV 2c
M8	5	f	Mixed	Gaborone	V	Not vac	Pos	21.12	CPV 2c
M9	5	f	Mixed	Gaborone	V	Not vac	Pos	21.61	CPV 2c
M10	3	m	Rhodesian Ridgeback	Gaborone	V,Di,De,A,F	Vacc	Neg	19.91	CPV 2c
M11	12	f	Jack Russel Terrier	Phakalane	Di,De,A,F	Par vacc	Pos	25.06	CPV 2c
M12	6	m	Jack Russel Terrier	Gaborone	V,Di,A,F	Par vacc	Neg	24.23	CPV 2c
M13	3	f	Boerboel	Phakalane	V, Di	Vacc	Pos	9.34	CPV 2c
M14	5	f	Mixed	Kgale	V,Di	Not vac	Pos	25.39	CPV 2c
M15	5	f	Jack Russel Terrier	Oodi	V,Di,De,A,F	Not vac	Neg	22.12	CPV 2c
M16	4	m	Great Dane	Phakalane	V,Di,De,A	Not vac	Pos	19.17	CPV 2c
M17	5	m	Boerboel	Mogoditshane	Di,A	Not vac	Neg	29.52	CPV 2c
M18	12	m	German Sheperd	Phakalane	V,Di,A,F	Not vac	Neg	32.19	CPV 2c
M19	2	m	Boerboel	Gaborone	V,Di,De,A	Vacc	Pos	21.18	CPV 2c
M20	2	m	Maltese	Phakalane	V,Di,De,A	Vacc	Pos	20.37	CPV 2c
M21	1.5	f	Tswana	Mmopane	V,Di,A	Vacc	Pos	14.80	CPV 2c
M22	7	f	Yorkie	Phakalane	V,Di,A,F	Vacc	Pos	12.00	CPV 2c
M23	5	m	Maltese	Phakalane	Di,De,A	Vacc	Neg	15.55	CPV 2c
M24	3	m	Dachshund	Phakalane	V,Di,A	Par vacc	Pos	17.13	CPV 2c
M25	2	m	German Sheperd	Phakalane	Di,A	Not vac	Pos	15.03	CPV 2c
M26	4	m	Dachshund cross	Phakalane	Di,De,A	Not vac	Neg	16.58	CPV 2c
M27	4	m	Dachshund cross	Phakalane	V,Di,De,A	Not vac	Pos	22.08	CPV 2c
M28	2	m	Maltese	Ruretse	V,Di,De,A,F	Not vac	Neg	26.09	CPV 2c
H1	2.5	f	Mixed	Tlokweng	V,Di,A,F	Not vac	Neg	22.90	CPV 2c
H2	2	f	Mixed	Tlokweng	V,Di,De,A,F	Not vac	Neg	34.86	CPV 2c
H3	2.5	m	Maltese	Tlokweng	V,Di,De,A,F	Not vac	Neg	16.74	CPV 2c
H4	3	f	Labrador	Tlokweng	V,Di,De,A,F	Vacc	Pos	11.40	CPV 2c
H5	3	m	Labrador	Tlokweng	V,Di,De,A,F	Par vacc	Pos	11.57	CPV 2c
H6	1.5	m	Maltese	Phakalane	V,Di,De,A,F	Not vac	Pos	15.39	CPV 2c
H7	3	f	Jack Russel Terrier	Tlokweng	V,Di,L,F	Vacc	Neg	13.12	CPV 2c
H8	6	m	Jack Russel Terrier	Gaborone	V,Di,A,M	Not vac	Pos	13.22	CPV 2c
H9	3	m	Boerboel	Gaborone	V,Di,A,F	Vacc	Pos	14.38	CPV 2c
H10	3	m	Mixed	Gaborone	V,Di,A	Not vac	Pos	12.82	CPV 2c

H11	4	f	Tswana	Tlokweng	Di,De,A	Vacc	Neg	14.19	CPV 2c
H12	3	m	Boerboel	Tlokweng	V,Di,De,A,F	Not vac	Neg	12.82	CPV 2c
H13	3	m	German Sheperd	Tlokweng	V,Di,De,A,F	Vacc	Pos	12.68	CPV 2c
H14	3	m	Mixed	Tlokweng	V,Di,De,A,F	Par vacc	Pos	18.04	CPV 2c
H15	2.5	m	Mixed	Tlokweng	V,Di,De,A,F	Par vacc	Pos	14.69	CPV 2c
H16	4	m	German Sheperd	Tlokweng	V,Di,De	Par vacc	Neg	31.39	CPV 2c
H17	3	m	Jack Russel Terrier	Tlokweng	V,Di	Par vacc	Neg	30.65	CPV 2c
H18	5	m	Tswana	Tlokweng	V,Di,De,A,F	Not vac	Pos	18.82	CPV 2c
H19	6	f	Jack Russel Terrier	Tlokweng	V,Di,De,A,F	Not vac	Neg	18.29	CPV 2c
H20	1.5	m	Maltese	Tlokweng	V,Di,De,A,F	Vacc	Pos	31.68	CPV 2c
H21	3	f	German Sheperd	Tlokweng	V,Di,De,A,F	Par vacc	Pos	15.21	CPV 2c
H22	4	m	Rottweiler	Tlokweng	V,Di,De,A,F	Par vacc	Pos	14.70	CPV 2c
H23	1.5	m	Jack Russel Terrier	Tlokweng	V,Di,De,A,F	Vacc	Pos	13.73	CPV 2c
H24	4	f	Rottweiler	Gaborone	V,Di,De,A,F	Not vac	Pos	16.96	CPV 2c
H25	1.5	m	Rottweiler	Tlokweng	V,Di,De,A,F	Not vac	Pos	12.86	CPV 2c
V1	4	f	Jack Russel Terrier	Gaborone	V,Di,De,A,F	Not vac	Pos	33.71	CPV 2c
V2	1.5	f	Maltese	Tlokweng	V,De,A	Not vac	Neg	12.65	CPV 2c
V3	3	Ff	Rottweiler	Gaborone	V,Di,De,A,F	Not vac	Pos	14.34	CPV 2c
V4	5	f	Jack Russel Terrier	Tlokweng	V,Di,De,A,F	Vacc	Neg	23.92	CPV 2c
V5	7	m	mixed	Gaborone	V,Di,De,A,F	Vacc	Pos	15.97	CPV 2c
V6	6	m	Mixed	Gaborone	No data	Vacc	Neg	21.08	CPV 2c
V7	6	m	mixed	Gaborone	No data	Vacc	Pos	15.72	CPV 2c
BC1	3	m	Boerboel	Gaborone	V,Di,De	Vacc	Pos	20.74	CPV 2c
BC2	3	m	Boerboel	Gaborone	V,Di,De	Par vacc	Pos	21.52	CPV 2c
BC3	7	f	Maltese cross	Gaborone	Di,De,A	Not vac	Pos	14.32	CPV 2c
BC4	7	f	Maltese cross	Gaborone	Di,De, A	Not vac	Pos	14.40	CPV 2c
J1	4	f	mixed	Maun	No data	Unknown	Neg	17.02	CPV 2a
J2	3	m	Boerboel	Maun	No data	Unknown	Neg	20.29	CPV 2c
J3	6	f	mixed	Maun	No data	Unknown	Pos	18.46	CPV 2c

NB: (Red=Samples submitted for sequencing) (V=vomiting, Di= Diarrhea, De= Dehydration, A= Anorexia, F=Foul smelling, Vacc=vaccinated, Neg=negative, Pos=positive)

Supplementary Table S2: Botswana CPV2 samples and their assigned accession numbers

Accession number	Strain	CPV variant	Country	Year
PX920305	G-12	CPV-2c	Botswana	2023
PX920306	BC-1	CPV-2c	Botswana	2023
PX920307	BC-2	CPV-2c	Botswana	2023
PX920308	G-3	CPV-2c	Botswana	2023
PX920309	G-32	CPV-2c	Botswana	2023
PX920310	G-37	CPV-2c	Botswana	2023
PX920311	G-41	CPV-2c	Botswana	2023
PX920312	G-50	CPV-2c	Botswana	2023
PX920313	H-10	CPV-2c	Botswana	2023
PX920314	S-16	CPV-2c	Botswana	2023
PX920315	S-21	CPV-2c	Botswana	2023
PX920316	S-30	CPV-2c	Botswana	2023
PX920317	S-53	CPV-2c	Botswana	2023
PX920318	S-6	CPV-2c	Botswana	2023
PX920319	S-11	CPV-2c	Botswana	2023
PX920320	M-27	CPV-2c	Botswana	2023
PX920321	G-35	CPV-2c	Botswana	2023
PX920322	H-11	CPV-2c	Botswana	2023
PX920323	H-12	CPV-2c	Botswana	2023
PX920324	M-2	CPV-2c	Botswana	2023
PX920325	J-1	CPV2a	Botswana	2023
